# Nano-level assay of attention-deficit/hyperactivity disorder medicament, atomoxetine by molecular-size-based resonance rayleigh scattering strategy. Employment in content uniformity, dosage form, and plasma analysis

**DOI:** 10.1186/s13065-023-01094-y

**Published:** 2023-12-06

**Authors:** Ahmed A. Abu-hassan

**Affiliations:** Department of Pharmaceutical Analytical Chemistry, Faculty of Pharmacy, Assiut Branch, Al-Azhar University, Assiut, 71524 Egypt

**Keywords:** Atomoxetine, Content uniformity, Plasma, Resonance rayleigh scattering, Spectrofluorimetric

## Abstract

The psychoanaleptic medication atomoxetine (ATX) is prescribed to cure attention-deficit hyperactivity syndrome. ATX works by selective prevention of norepinephrine reuptake. It acts by raising the brain’s natural level of norepinephrine, which is necessary for behavior regulation. In this study, a sensitive and practical experimental method was employed to analyze the presence of ATX. The approach utilized a green chemistry-compatible technique, known as a one-pot experiment. The main principle behind this method was the use of molecular-size-dependant resonance Rayleigh scattering (RRS) phenomenon, which occurred due to the interaction between the dual complex of Cilefa Pink B and ATX. When ATX medication and Cilefa Pink B were combined in an acidic environment, they formed an association complex, leading to an amplification of the RRS signal. This amplification directly correlated with the concentration of ATX, specifically within the range of 40-1250 ng/mL. The RRS signal was monitored at a wavelength of 352 nm. The sensitivity of the method was demonstrated by the determination of the limit of detection (LOD) at 12.9 ng/mL and the limit of quantitation (LOQ) at 39.2 ng/mL. The variables of the method were thoroughly investigated and optimized. To ensure the reliability of the method, it was validated according to the International Council for Harmonisation (ICH) guidelines. Furthermore, the method was successfully applied to analyze ATX in its prescribed dosage form. The achievement of using the established resonance Rayleigh scattering (RRS) technology to analyze the target drug in plasma and ensure content uniformity was a remarkable feat.

## Introduction

Atomoxetine HCl has the chemical name (R) n-methyl-3-(2-methylphenoxy)-3-phenyl propylamine hydrochloride, and M.Wt of 291.8 g/mole. ATX is a psychoanaleptic, and it works by specifically inhibiting norepinephrine reuptake. The stated medication is used to treat attention deficit hyperactivity disorder (ADHD). The most prevalent neurobehavioral condition of childhood is ADHD, and some individuals’ symptoms last into adulthood. It is characterized by signs of impulsivity, hyperactivity, and inattentiveness that interfere with social and academic performance [1–3].

Various analytical methods have been investigated for the analysis of ATX, including electrochemical [4, 5], high-performance liquid chromatography [6–9], gas chromatography [10], high-performance thin-layer chromatography [11], capillary electrophoresis [12], and spectrophotometry [13–15]. The fluorimetric techniques for ATX analysis have been limited [2, 16]. However, the technique offers significant advantages such as intelligent selectivity, exceptional sensitivity, minimal solvent consumption, availability in most laboratories, rapid analysis time, and ease of instrument use. These advantages motivated us to develop a straightforward, environmentally friendly, and sensitive ATX testing approach that would outperform the previously published method which utilizes 4-chloro-7-nitro-2,1,3-benzoxadiazole.

To evaluate an extensive range of pharmaceutical drugs, a molecular-size-based resonance Rayleigh scattering probe (MSB-RRS) was created, employing different chemical xanthene dyes and pigments as probes. This was achieved by forming ion association complexes. Some of these dyes possess a bulky structure that naturally emits fluorescence. Under specific pH conditions, the dye can undergo ionization and form association complexes with the target analyte.

This modification can result in different outcomes such as generating a new color [17], suppressing the natural fluorescence of the dye [18, 19], or enhancing the RRS signal of the dye [20–22]. These phenomena can be utilized in the quantification of the target analyte. However, when it comes to detecting medication at the nanogram level, Resonance Rayleigh scattering-based methods are typically employed. The simplicity and high sensitivity of the RRS strategy make it widely applicable in various fields, including nucleic acids [23], glycosaminoglycan [24], proteins [25], drug efficacy [26], metal ions quantification [27], and organic substances [28].

Resonance Rayleigh scattering is a valuable technique that is widely used in the field of pharmaceutical analysis for the determination of drugs. This innovative method utilizes the scattering of light to accurately measure the concentration of drug compounds in a sample solution. By exploiting the unique physical properties of the drug molecules and their interactions with incident light, resonance Rayleigh scattering provides a sensitive and precise means of drug quantification. One of the key advantages of resonance Rayleigh scattering is its ability to work with low concentrations of drugs, making it highly useful in pharmaceutical research and development. The method relies on the principle that when light interacts with drug molecules in a sample, it scatters in a particular manner that is characteristic of the particles present. Through careful analysis of the scattered light, scientists can deduce vital information about the drug’s concentration and structural properties. Moreover, the technique offers several benefits over traditional methods of drug determination. Resonance Rayleigh scattering is non-destructive, meaning that the sample remains intact throughout the measurement process. This is particularly advantageous when working with valuable or limited samples. Additionally, the method is highly sensitive, and capable of detecting even minute concentrations of drugs. Furthermore, resonance Rayleigh scattering is relatively simple and cost-effective, requiring minimal equipment and expertise.

Cilefa Pink B (CPB) which belongs to xanthene dye plays a crucial role in drug assays and is widely utilized. In an acidic environment, the amino group (secondary) of ATX becomes protonated, facilitating the formation of an ion-pair complex between CPB and ATX. This resulting “on-on + RRS system” is employed for ATX quantification, as the enhancement in Resonance Rayleigh Scattering is directly proportional to the concentration of ATX. This method is considered cost-effective due to the affordability of the chemicals involved and the ready availability of the required equipment in quality control laboratories.

The objective of the study is the employment of RRS for the determination of ATX. Developing an eco-friendly, direct, fast, simple, and sensitive size-dependent spectroscopic method for examining the considered drug, ATX, has become critical. The developed size-dependent spectroscopic method can accomplish these goals by reacting the medication under study with self-fluorescent dyes in an acidic solution. As a result, ATX could be swiftly analyzed for its concentration in bulk forms, medicinal preparations, and biological fluids. The spectrofluorometer is adjusted at synchronous mode using Δλ = zero. The scanned spectrum is developed first and the selection of the wavelength provokes the highest ΔRRS for approach development. All variables affecting approach development are studied and optimized. The proposed approach has been thoroughly validated under ICH requirements.

## Experimental

### Apparatus

An SCINCO spectrofluorometer FS-2 from Korea, equipped with a 150 W Xe-arc lamp, served as the light source for fluorescence intensity (FI) monitoring. Reagents and ATX powder were weighed using a single-pan electronic balance (XB 220 A Precisa, Switzerland). The pH of the solution was adjusted using a calibrated pH meter (Milwaukee MW101). Additionally, an Ikon Ultrasonic Bath and a thermostatic water bath were utilized in the experimental setup.

### Material and reagents

The ATX powder (99.92% purity) was generously provided by Mash Premiere for Pharmaceutical Industries located in Badr city, Egypt. Atomox apex 40 mg capsules and Strattera 18 mg were purchased from a local pharmacy in Assuit. The xanthene dye was obtained from Sigma Aldrich in the USA. Organic solvents, citric acid, NaOH, and phosphoric acid were procured from El-Gomhoria Co in Tanta, Egypt.

### Reagents and solutions

The Teorell buffer was utilized in the development of the strategy due to its ability to cover a broad pH range from 2 to 12. It consists of two main solutions: solution I, which is 0.1 M HCl, and solution II, which constitutes 3.5 mL phosphoric acid, 343 mL sodium hydroxide (1 M), and 100 ml of citric acid (0.33 M) in 1000 mL flask that filled to the limit with water. CPB (0.02 W/V) was prepared be dissolving 20 mg of CPB in 100 mL of distilled water. The desired pH for the experiment is achieved by mixing appropriate quantities of these two solutions (I&II) [29–31]. To achieve a desired concentration of 200 µg/mL of ATX, 20 mg of ATX was sonicated in 30 mL of water using a labeled calibrated flask. The volume was then increased to 100 mL with purified water. Further dilutions with water were performed to make necessary adjustments as required for the experiment.

### Procedures for implementing experiments

To initiate the investigation on resonance Rayleigh scattering (RRS), we prepared a series of calibrated flasks (10 mL capacity) containing specific amounts of the ATX standard solution. The concentrations of the solutions ranged from 50 to 1300 ng/mL. Each flask was then supplemented with 1.2 mL of Teorell regulating buffer (pH = 3.1) and 1.2 mL of CPB (0.02 W/V). The contents of each flask were gently mixed and subsequently combined with distilled water. After vigorous mixing, the solutions were set aside for an additional five minutes to allow for aging. By employing synchronous spectrofluorimetry with an excitation wavelength of 352 nm, we monitored the impact of ATX on the RRS amplitude of the dye. The obtained RRS readings were normalized against the results of a blank experiment, which was conducted simultaneously with each measurement. Blank experiments are carried out separately and anlyte is omitted under the same experimental conditions. The purpose of the analytical blank experiment is to identify and quantify any sources of contamination or interference that may affect the accuracy and integrity of the analytical results. By comparing the measurements obtained from the blank experiment to those of the sample, analysts can assess and correct for any background noise or contamination, ensuring the reliability of their analytical measurements.

### Steps for ATX assay in capsules and testing their uniformity

To begin, 10 capsules of either Atomox apex 40 mg or Strattera 18 mg were emptied and the precise amount of ATX content, equivalent to 20 mg, was carefully transferred into a calibrated jar with a capacity of 100 mL. The powder was thoroughly mixed and sonicated with 60 mL of water for 15 min. Then, the same solvent was added to reach the mark on the jar. The solution was then clarified, and the resulting filtrate was collected in another calibrated jar of the same capacity. This process involved discarding the initial amount of filtrate, and further dilution was done to obtain an operational solution with ATX concentrations falling within the desired linearity range of the assay. The outcomes of sequential operational experiments were evaluated. To ensure the uniformity of ATX in Strattera 18 mg capsules, the aforementioned steps were repeated with ten capsules, but each individual assay used a single capsule.

### Steps for ATX assay in plasma

This study was performed depending on the ethical committee of Al-Azhar University with approval No. AZ/AS/PHREC/33/2023, and informed assent was gotten for any tests with humans.

Blood samples obtained from Egyptian humans were processed and used in the ATX assay. A volume of 1 mL of frozen plasma obtained after isolation was added to a series of 10 mL tubes. 0.5 mL of ATX at the desired concentration was mixed with the plasma, and then 3.5 mL of acetonitrile was added. The content of the tubes was vortexed for 15 min to separate the plasma proteins. The resulting solution, which fell within the linearity range, was subjected to complexation and measurements following the general assay steps.

## Result and discussion

Ion pair complex formation refers to the process in chemistry where two oppositely charged ions associate with each other to form a stable complex. This interaction occurs when an ion with a positive charge (cation) and an ion with a negative charge (anion) come together and form a close association due to electrostatic forces. Ion pair complex formation plays a significant role in various chemical processes, including organic synthesis, pharmaceutical reactions, and some analytical techniques. Xanthene-based dyes have emerged as a crucial avenue for spectroscopic studies due to their strong inherent fluorescence and the potential for forming complex associations with various medicinal compounds. These dyes offer a wide range of spectroscopic approaches, including colorimetry [17], spectrofluorometry [18, 19], and RRS [20, 21]. The use of these substances has led to various interesting features such as the generation of new colors, suppression of native dye fluorescence, and amplification of RRS peaks. Examples of xanthene-based dyes include fluorescein, eosin, and Califa Pink B.

**Fig. 1:**
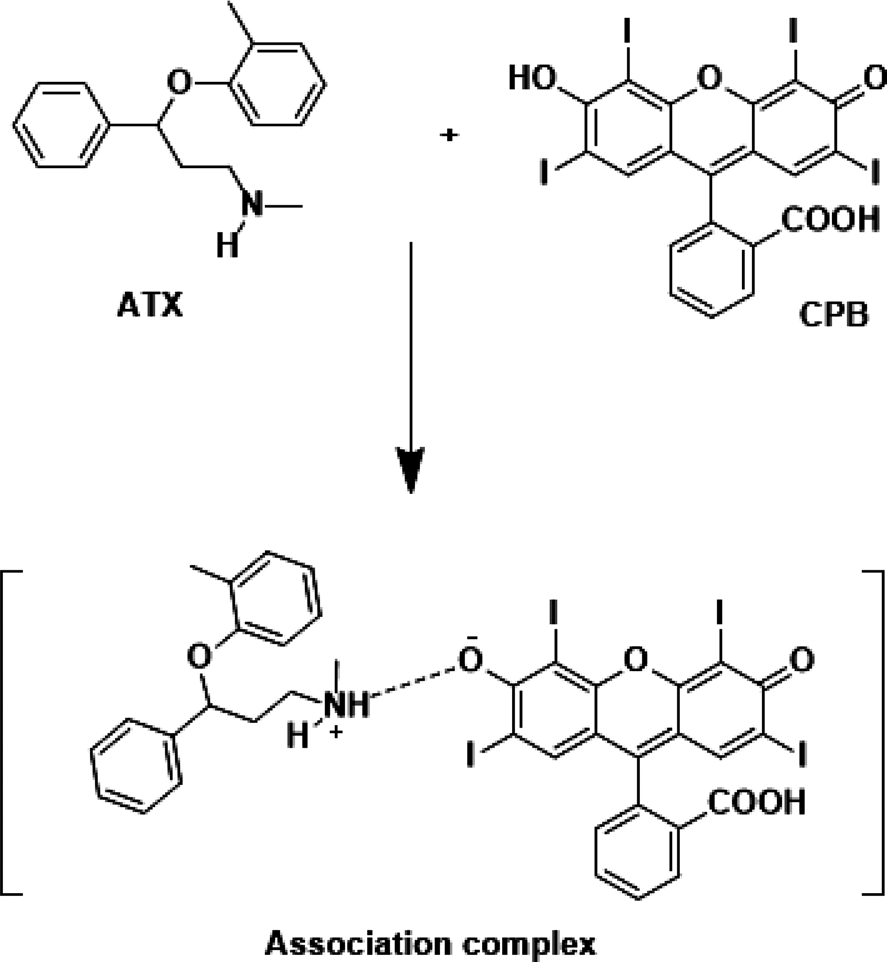
Representation of association complex formation

**Fig. 2:**
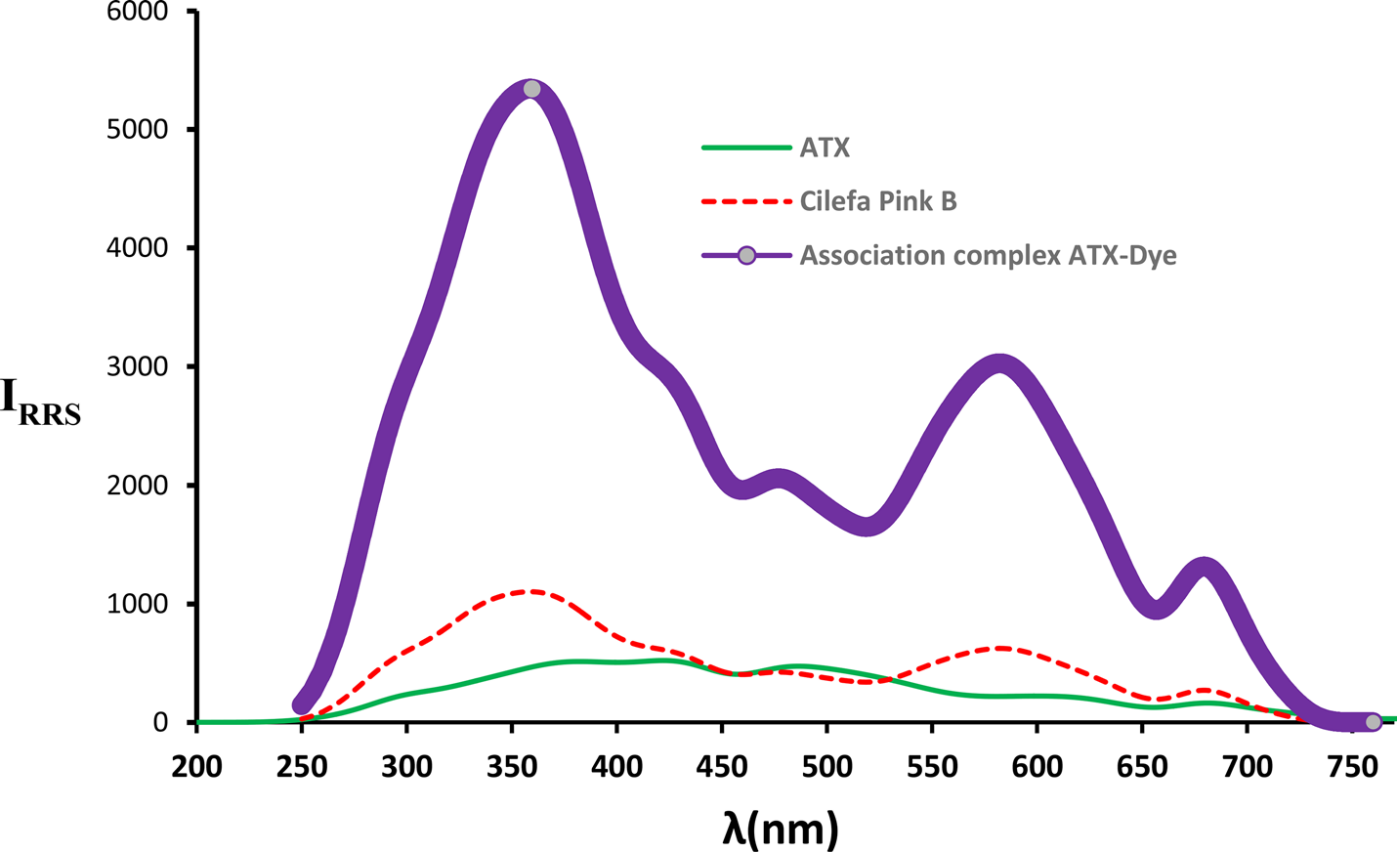
RRS spectrum of cilefa pink (0.02 W/V), ATX (750 ng/mL), and the formed association complex ATX-Dye

The described methodology utilized Califa Pink B for the analysis of ATX using RRS. In an acidic environment, the secondary amino group of ATX becomes protonated, allowing for the formation of an ion-pair complex between CPB and ATX (Fig. 1). The resulting “on-on + RRS system” is directly proportional to the ATX concentration, enabling the quantification of ATX. Figure 2 illustrates the RRS of the drug alone (green line), cilefa pink (red line), and the formed complex (violet color). As shown there is a significant increase in the RSS accompanied complex formation. This procedure is considered both safe and cost-effective due to the affordable chemicals involved and the regular availability of necessary equipment in quality control laboratories.

### RRS mechanism

#### Molecular volume impact

The primary condition for intensification of the RRS response is an increase in volume brought on by an increase in the incorporated molecules in the dual associate complex. The study is known as molecular size-based as a result. Since it is difficult to get an idea of the molecule volume, this formula can be used as a substitute to explore this requirement [32].

$$\mathbf{I} = \mathbf{K}\mathbf{C}\mathbf{M}\mathbf{I}^\circ$$

where I represents RRS strength, K coefficient constant, M is the M.Wt, I the incident intensity, and C refers to solution concentration.

In the case where the variables in the previous equation are kept constant, the RRS amplitude will be directly proportional to the molecular weight of the integrated molecules. In the current reaction, the weight of the ATX-Dye complex (C_37_H_29_I_4_NO_6_) increases from 256.16 (protonated ATX) to 1090.82 g/mol, which will result in an intensification of the RRS band.

#### Hydrophobic interface creation

The development of hydrophobic interfaces is closely linked to the signal that RRS produces [33]. prior to ion interaction required to produce the complex ATX is protonated (cationic form), whereas CPB is present in anionic form. The hydrated form of both is more noticeable in this instance. When these ions combine to form a complex, hydrophobicity rises, which enhances RRS. The neutralization of ion charges and the repulsion field produced by counter ions of non-polar groups are related to the improvement of hydrophobicity [34].

#### Molecular rigidity and planarity

Molecular rigidity refers to the inability of a molecule to undergo significant conformational changes or bond rotations. This can arise from the presence of strong intramolecular interactions. A rigid molecule maintains a fixed shape or conformation, even when subjected to external forces or changes in its environment. Planarity, on the other hand, refers to the arrangement of atoms in a molecule where they lie in the same plane or nearly the same plane. Molecular rigidity and planarity often go hand in hand, as planar molecules tend to be more rigid due to the restrictions imposed by strong covalent bonds or extended pi-systems. The research listed below [32, 35, 36] demonstrates that the development of association complexes increases rigidity and restricts the movement of the aryl group, resulting in altered planarity, all of which are risk factors for RRS amplification.

### Adjusting the variables of the study

For the highest RRS performance score, the authors looked into every aspect of the trial. Each variable’s value was changed while using the fixed values of other variables to conduct numerous tests.

#### pH and buffer volume

**Fig. 3:**
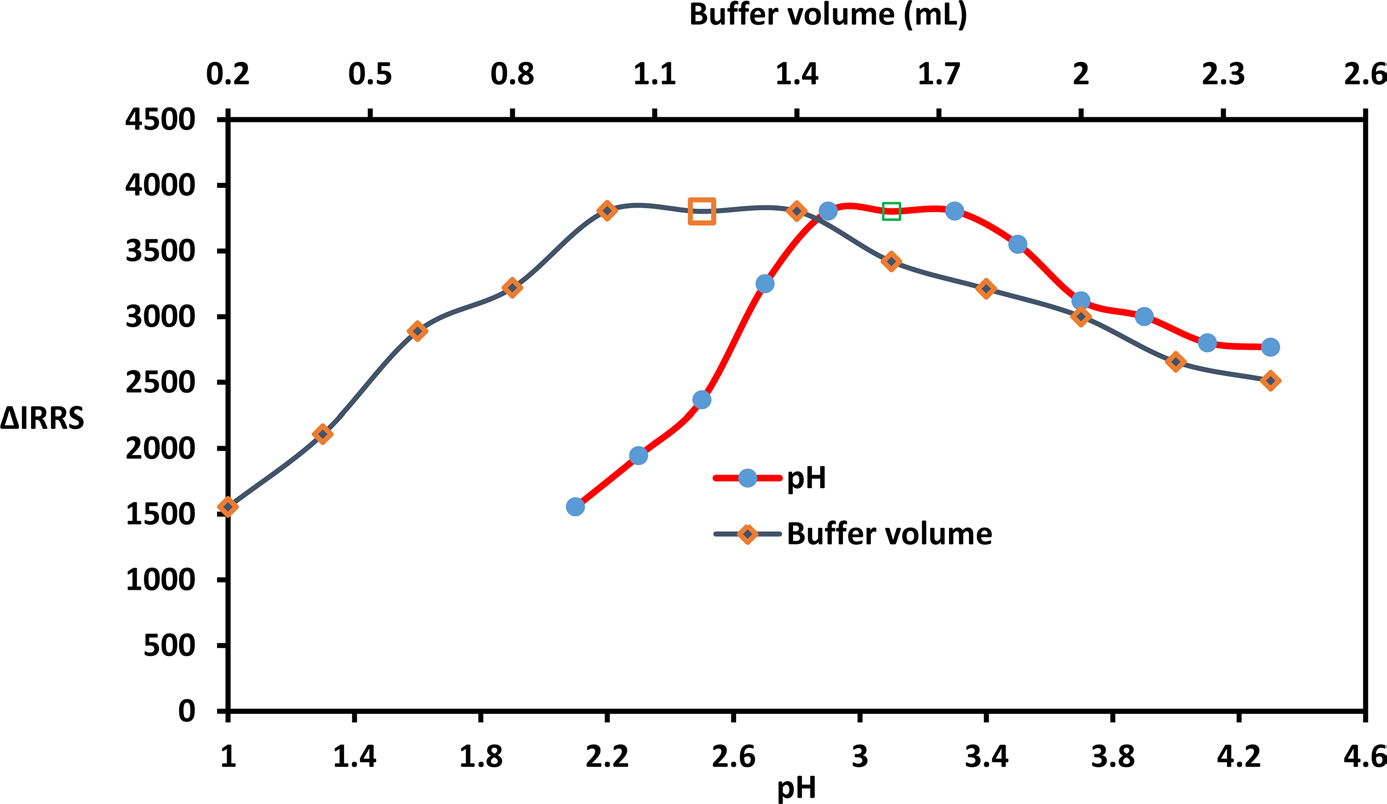
Impact of buffer volume and pH on the suggested system

The provided methodology investigated the influence of acidic pH on the complexation between ATX and CPB. The pH of the environment significantly affects the formation of the ATX-dye complex. The highest RRS score is observed within the pH range of 2.9–3.3. Beyond pH 3.3, the RRS score gradually decreases. In this study, the optimal pH was determined to be 3.1 (Fig. 3). Additionally, the impact of different volumes of Torell buffer on the complexation between ATX and CPB was examined. The maximum RRS signals were observed within the range of 1.0-1.4. Any volume lower or higher than these limits resulted in a considerable decrease in the corresponding RRS score. This can be attributed to insufficient buffer volume’s inability to effectively change the pH or an increased salt concentration, which hinders complex formation by stabilizing the charged ions. Consequently, a buffer volume of 1.2 was considered suitable for this research (Fig. 3).

#### Amount of CPB reagent

**Fig. 4:**
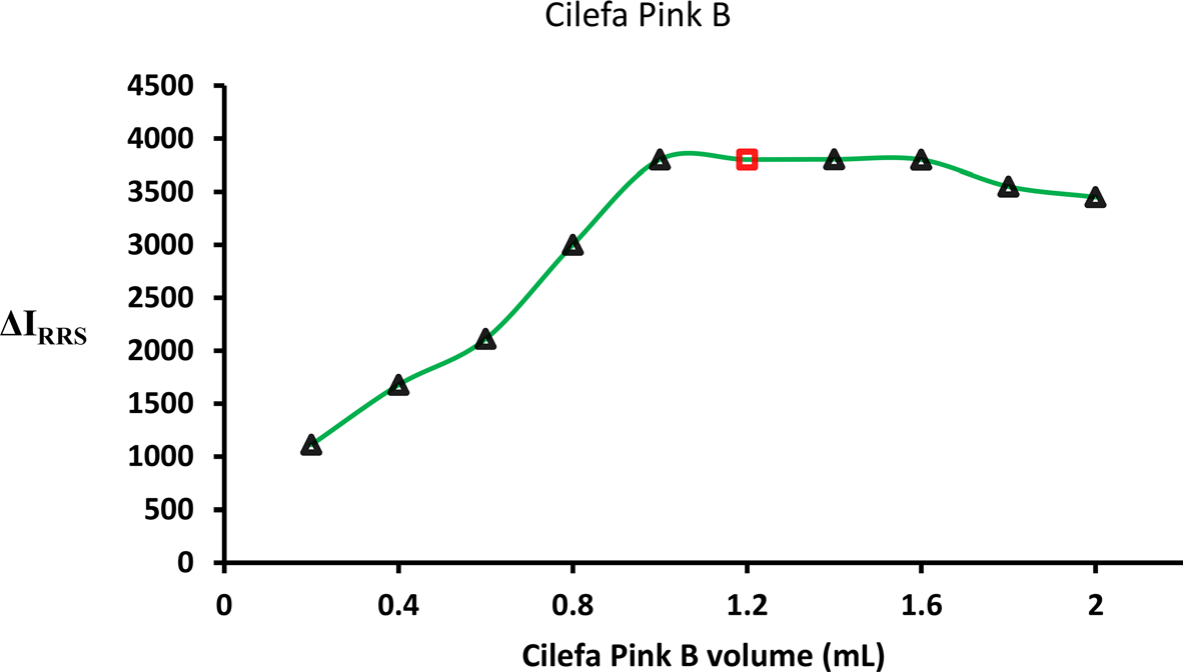
Impact of various volumes of CPB (0.02 W/v) on the proposed methodology

In the current experiment, the CPB reagent was utilized at various concentrations. A stock solution of CPB (0.02 W/V) was initially prepared, and different volumes (ranging from 0.2 to 2.0 mL) of this solution were used in the assay. After each experiment, the RRS signal was recorded. It was observed that smaller volumes of the stock solution (0.2–0.8 mL) gradually improved the RRS signal, and the maximum RRS response was achieved within the volume range of 1.0 to 1.6 (Fig. 4). However, after this point, a decrease in RRS signal was noticed, which may be attributed to the tendency of CPB to self-aggregate.

#### Dispersing solvent and time impact

**Fig. 5:**
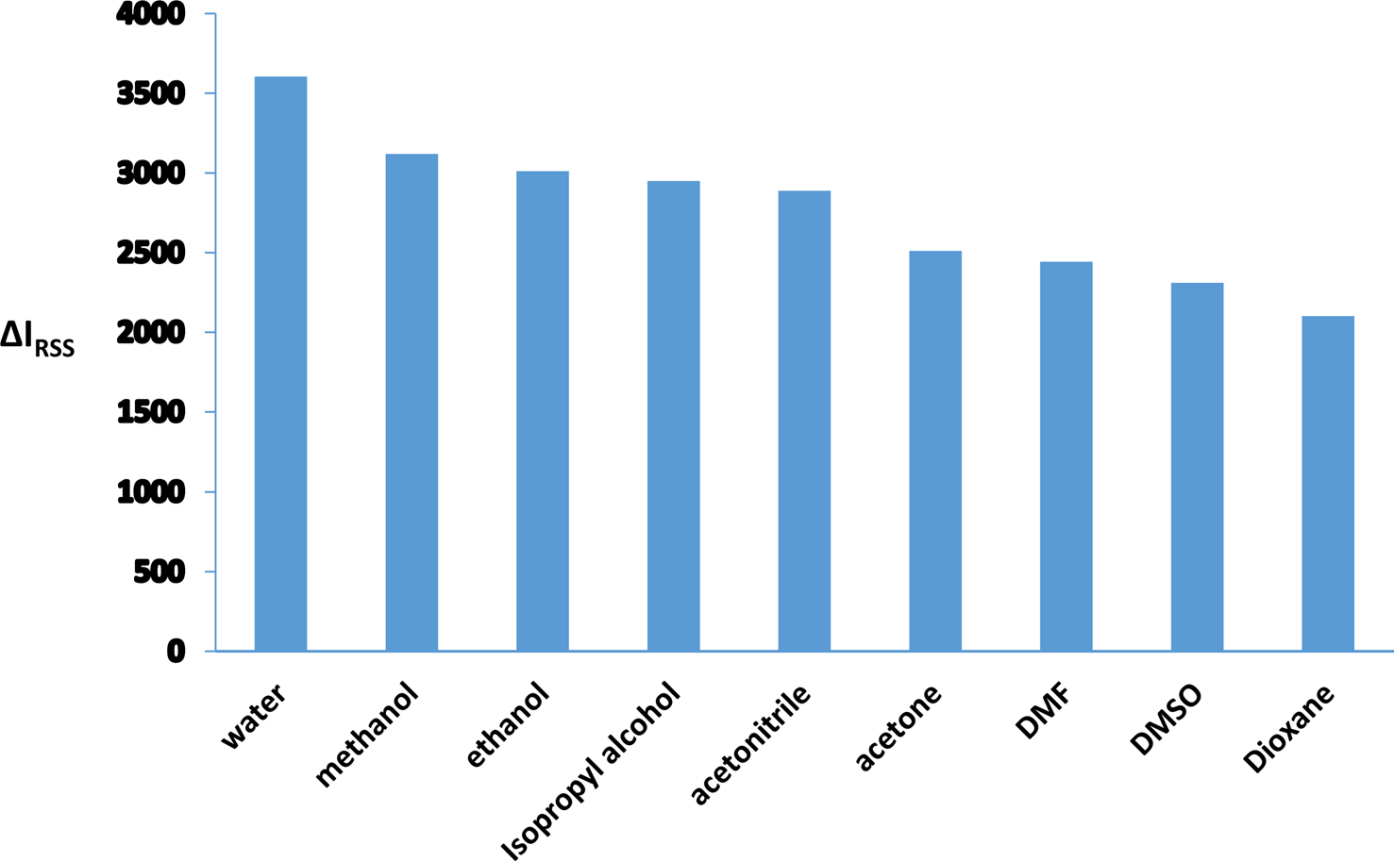
Impact of various diluting solvents on RRS intensity of the formed complex

The study experimented with various dispersing mediums, including water, alcohols (ethyl, isopropyl, methyl), acetonitrile, DMF, acetone, and Dioxane. The use of water yielded the best results in terms of improving RRS (Fig. 5). Water, being a green solvent with a high polarity index (around 9) and a dielectric constant of 80.2, facilitated the formation of the association complex easily and effectively. The unsatisfactory outcomes observed with other organic solvents may be attributed to their potential to degrade the quality of the final complex. These solvents have the potential to alter or denature the stability of the complex system. It is worth noting that the chosen system is completely miscible in water due to the water-solubility of its constituents. However, low miscibility may occur with other organic solvents due to differences in their dielectric constants, which can make the formation of the complex system more challenging.

### Method validation

The method was validated according to ICH guidelines [37–39]. The validation parameters include accuracy, precision, linearity and range, selectivity, LOQ, LOD, and robustness.

#### Linearity, range, LOQ, and LOD

The developed RRS analysis technique was used to investigate typical ATX solutions. A calibration graph was created by plotting the difference in RRS against ng/mL concentrations of ATX. The RRS methodology exhibited a linear response within a range of concentrations (40-1250 ng/mL), showing remarkable correlation coefficients. The calibration was expressed by a regression equation (Y = 3.085 X + 1494.3, r = 0.9998), and the corresponding data were presented in Table 1. Sensitivity parameters such as LOQ (Limit of Quantification) and LOD (Limit of Detection) were analyzed based on the ICH (International Council on Harmonisation) equations.

**Table 1:** Information on the developed system’s statistics

Parameters	Values
Linear range (ng/mL)	40-1250
Slope	3.085
Intercept	1494.3
SD of intercept (S_a_)	12.1
Correlation coefficient (r)	0.9998
Determination coefficients (r^2^)	0.9997
Number of determinations (3 replicates for each)	8
Limit of quantitation (ng/mL)	39.2
Limit of detection (ng/mL)	12.9

SD is the standard deviation

$$\varvec{L}\varvec{O}\varvec{Q}=\frac{10 \sigma }{Slope} , \varvec{L}\varvec{O}\varvec{D}=\frac{3.3 \sigma }{Slope}$$

where 1D70E refers to the standard deviation of the intercept.

Due to the high molecular weights of both the medicinal compound and dye, the methodology showed an elegant level of sensitivity.

#### Accuracy and precision

Experiments were conducted on the planned technology to assess the spectroscopic accuracy. Four different content levels (200, 500, 900, and 1100 ng/mL) were used in the analysis. The accuracy of the RRS spectroscopic analysis technique was evaluated using % error and percentage recovery as metrics. The results confirming the accuracy of the technique are presented in Table 2. Furthermore, the developed technique was employed to analyze standard drug materials at three different tested doses (400, 750, and 900 ng/mL) in order to evaluate the inter-day and intra-day precisions. The precision of the proposed RRS technique was determined by calculating the RSD (Relative Standard Deviation). The RSD values at both low and intermediate levels were found to be acceptable, being less than 2%, as shown in Table 3.

**Table 2:** Accuracy of the developed technique at four ATX concentrations

Conc. level (ng/mL)	Recovery^*^± SD	% Error
200	99.20 ± 1.23	0.80
500	98.26 ± 0.65	1.74
900	98.88 ± 1.08	1.12
1000	101.38 ± 1.67	1.38

*mean of three ATX determinations and SD is the standard deviation

**Table 3:** Precision of the current approach at two levels

Conc. Level (ng/mL)	Intraday precision		Interday precision	
	Recovery^*^± SD	% RSD	Recovery^*^± SD	% RSD
300	100.63 ± 1.25	1.24	99.23 ± 1.19	1.19
750	98.89 ± 1.32	1.34	100.60 ± 1.65	1.64
950	100.85 ± 1.02	1.01	101.08 ± 1.38	1.36

*mean of three ATX determinations and RSD is the relative standard deviation

#### Robustness

The resilience of the method was evaluated by making small but significant changes to critical procedure-related variables, such as the solution’s pH (within a selected range of ± 0.1) and the volume of buffer and dye (within a selected range of ± 0.1 mL). The reliability of the procedure was assessed by calculating the recovery percentage standard deviation (% SD). The results showed that these slight changes did not have a dramatic impact on the RRS readings, as demonstrated in Table 4, indicating the durability of the applied approach.

**Table 4:** Evaluating the robustness of the existing ATX assay technology

Parameter	± optimum value	% Recovery* ± SD
pH	3.0	100.08 ± 0.35
	3.2	99.35 ± 1.28
Buffer volume (mL)	1.1	98.76 ± 1.18
	1.3	99.27 ± 0.71
Dye volume (mL)	1.1	98.37 ± 1.39
	1.3	101.02 ± 1.62

*Mean of three replicate measurements, SD = Standard deviation

#### Selectivity

To verify the effectiveness of the suggested methods, the impact of common capsule ingredients was evaluated through the standard addition method. The experiment included glucose, lactose, starch, talc, magnesium stearate, Macrogol (PEG 400), and sorbitol. Table 5 indicates that these ingredients do not cause any interference, as evidenced by the close-to-100% recovery rate and low standard deviation. This can be attributed to the absence of amino group in these compounds, which is necessary for the formation of ion pair complex.

**Table 5:** Assessment of the selectivity for the suggested method in the presence of some common excipients

Excipients	Amount added (µg mL^− 1^)	Drug con. (ng mL^− 1^)	% Recovery ± SD^a^
Mg stearate	10	550	100.53 ± 1.02
Starch	50	550	101.69 ± 0.62
Lactose	10	550	99.15 ± 0.90
Glucose	10	550	98.43 ± 1.56
Talc	5	550	98.35 ± 0.82
Sorbitol	10	550	99.21 ± 1.16
Macrogol (PEG 400)	30	550	101.02 ± 1.62

## Application

### Dosage form and homogeneity of capsules

The content of commercially available ATX formulations, specifically Atomox apex and Strattera, sold in Egyptian pharmacies, was analyzed using the provided approach. The same dose formulations were also examined using the approach described in reference [16]. The percentages of recovery for ATX formulation assay were compared between the current process and the published spectroscopic strategy using two statistical metrics (F-test and t-test). The results showed no substantial difference in accuracy and precision criteria between the developed process and the published strategy, as the t- and F-test values were lower than the tabulated scores (Table 6).

**Table 6:** Dosage form analysis of Atomoxetine and comparison with the reported method

Dosage form	Current method	Reported method	t- test value^a^	F-value^a^
	Recovery* ± SD	Recovery* ± SD		
Atomox apex 40 mg capsules	100.86 ± 0.88	100.39 ± 1.80	0.53	4.2
Strattera 18 mg capsules	98.75 ± 1.28	98.98 ± 1.68	0.25	1.70

^*^The value is the average of five determinations for both the proposed and reported methods

^a^Tabulated values at 95% confidence limit are t = 2.306, F = 6.338

When the concentration of the medicine in the capsule is below 25 mg or constitutes less than 25% of the capsule’s components, it is recommended to test the uniformity of the drug within the capsules. However, this process of examining each capsule’s content for homogeneity requires a significant amount of time and effort. As a result, the current procedure offers the advantages of being simple and quick, as it does not require time-consuming extraction or heating for the complex to be generated instantly. These advantages allow for the direct examination of Strattera 18 mg capsules. The data in Table 7 demonstrates that the homogeneity of Strattera 18 mg capsules is confirmed, as the acceptance value is lower than the maximum permitted value.

**Table 7:** Checking content uniformity of Strattera 18 mg capsules by the current approach

Capsule number	%Recovery
1	100.35
2	102.08
3	99.29
4	98.35
5	97.58
6	99.25
7	102.06
8	97.91
9	103.43
10	101.65
Mean ($\overline{X}$) ± S.D	100.20 ± 2.02
Acceptance value (AV)	4.85
Max. allowed AV (L1)	15

### Spiked plasma

The method’s sensitivity makes it suitable for measuring ATX in human plasma. Thus, THE created complex was used to track the final ATX doses in the bio samples. The developed approach was used to assess four separate plasma samples that had been tainted with ATX standard-dosed solutions. Table 8 demonstrates the tolerable SD and reasonable rate of recovery of the suggested method for estimating ATX concentrations in spiked plasma.

**Table 8:** Outcomes for ATX assay in spiked plasma

Conc. level (ng/mL)	Recovery*± SD
200	98.55 ± 2.11
500	97.83 ± 1.35
900	98.83 ± 1.01
1000	97.63 ± 1.35

*Mean of three ATX replicate measurements, SD = Standard deviation

#### Method greenness

Green chemistry is of great concern in analytical chemistry. According to the Environmental Protection Agency, Green chemistry is defined as chemistry that designs chemical products and processes that are harmless to the environment and human health, thus preventing pollution formation. Green chemistry can be achieved through several dominant trends, among them finding and testing new alternative, non-toxic, and renewable reaction media such as water, ionic liquids, and supercritical fluids. Therefore, a green and environmentally safe instrumental methodology is applied to determine ATX rapidly.

**Fig. 6:**
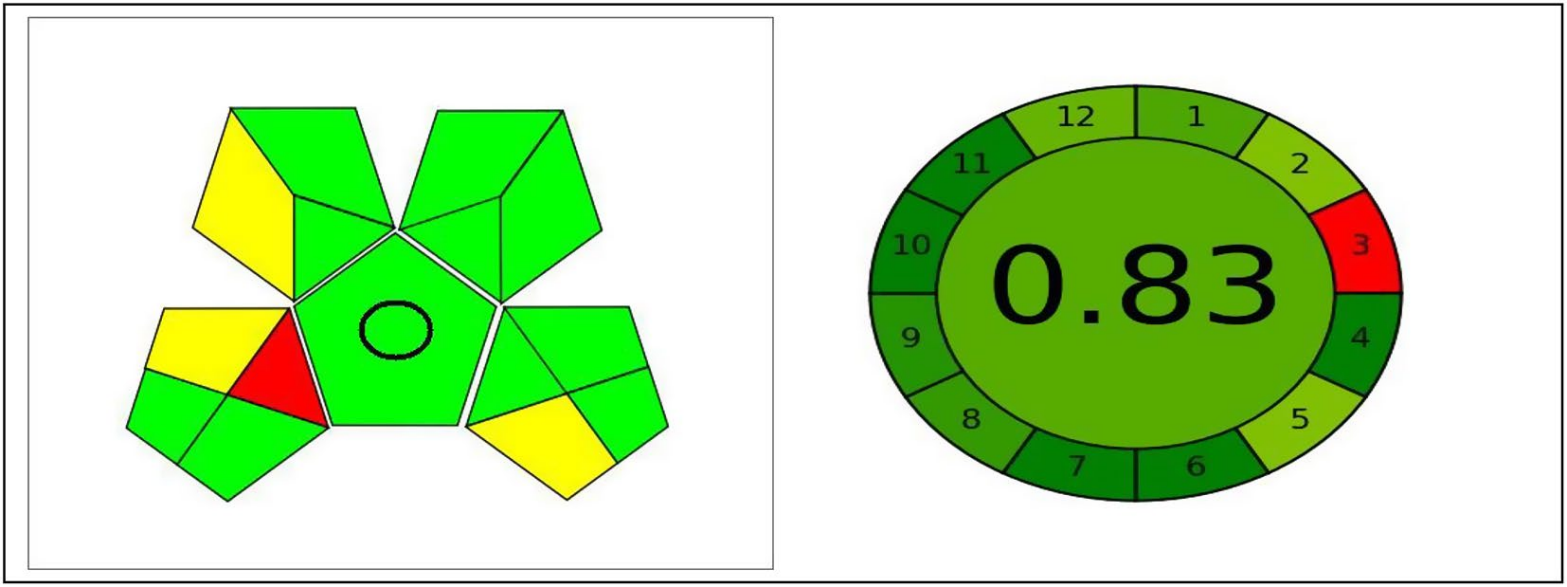
The greenness evaluation of the planned method using different ecological metrics

Numerous assessment tools have recently emerged to evaluate the ecological effects of different analytical methods. Assessing these methods is crucial in order to minimize the environmental pollution caused by these processes. To illustrate, a typical HPLC system generates approximately 0.5 L of organic waste every day [40, 41]. As a result, conducting a greenness assessment has become an essential evaluation. AGREE shows a clock-shaped pictogram, in which the perimeter is divided into 12 sections, each corresponding to a GAC principle. The center of the pictogram shows a numerical value estimating the ecological impact, where the closer to 1, the better the impact. As shown in Fig. 6 AGREE pictograms show the lowest ecological impact, as expressed by the numerical evaluation. GAPI system has five pentagrams. Pentagrams show the environmental impact of each analytical step. Environmental impact increases when green turns yellow, then red. When using the proposed method for pharmaceutical dosage form analysis, the method would be totally green due to the absence of any required organic solvents. The use of low-energy spectrofluorometric equipment, its higher throughput, and simple sample preparation procedures without the need for derivatizing agents account for better environmentally friendly behavior as described in Fig. 6.

## Conclusion

This research aimed to develop a novel and practical fluorimetric method for accurately measuring ATX in its various dosage forms and plasma. The study offers several advantages, including a simple and cost-effective single-container design, high sensitivity, and the elimination of the need for sample extraction. Compared to other analytical tools, this approach is recommended and advocated for its suitability in assessing ATX using a spectrofluorimeter. Spectrofluorometers are particularly attractive due to their widespread availability, affordability, selectivity in monitoring, ease of operation, and the fact that they eliminate the time-consuming process of sample pre-treatment. ATX, ranging from 40 to 1250 ng/mL, was detected as an ion pair through electrostatic attraction in a slightly acidic environment. The use of the CPB reagent poses minimal risks compared to other methods. The method successfully identified ATX in different forms, including genuine pharmaceutical and bio-spiked samples. As a result, this method can effectively evaluate treatment efficacy in laboratory settings, clinical trials, and pharmaceutical companies.

## Data Availability

The data will be available upon reasonable request from the corresponding author.
